# Ensemble-based classification approach for micro-RNA mining applied on diverse metagenomic sequences

**DOI:** 10.1186/1756-0500-7-286

**Published:** 2014-05-06

**Authors:** Sherin M ElGokhy, Mahmoud ElHefnawi, Amin Shoukry

**Affiliations:** 1 Department of Computer Science and Engineering, Egypt-Japan University of Science and Technology (E-JUST), 21934, New Borg El-Arab, Alexandria, Egypt; 2 Informatics and Systems Department and Biomedical Informatics and chemo informatics group, Division of Engineering Research and Centre of Excellence for Advanced Sciences, National Research Centre, Cairo 12622, Egypt; 3 Computer and Systems Engineering Department, Alexandria University, Alexandria, Egypt

**Keywords:** MicroRNA, Support vector machine, Random forests, MiRNA hairpin prediction, Neural network

## Abstract

**Background:**

MicroRNAs (miRNAs) are endogenous ∼22 nt RNAs that are identified in many species as powerful regulators of gene expressions. Experimental identification of miRNAs is still slow since miRNAs are difficult to isolate by cloning due to their low expression, low stability, tissue specificity and the high cost of the cloning procedure. Thus, computational identification of miRNAs from genomic sequences provide a valuable complement to cloning. Different approaches for identification of miRNAs have been proposed based on homology, thermodynamic parameters, and cross-species comparisons.

**Results:**

The present paper focuses on the integration of miRNA classifiers in a meta-classifier and the identification of miRNAs from metagenomic sequences collected from different environments. An ensemble of classifiers is proposed for miRNA hairpin prediction based on four well-known classifiers (Triplet SVM, Mipred, Virgo and EumiR), with non-identical features, and which have been trained on different data. Their decisions are combined using a single hidden layer neural network to increase the accuracy of the predictions. Our ensemble classifier achieved 89.3% accuracy, 82.2% f–measure, 74% sensitivity, 97% specificity, 92.5% precision and 88.2% negative predictive value when tested on real miRNA and pseudo sequence data. The area under the receiver operating characteristic curve of our classifier is 0.9 which represents a high performance index.

The proposed classifier yields a significant performance improvement relative to Triplet-SVM, Virgo and EumiR and a minor refinement over MiPred.

The developed ensemble classifier is used for miRNA prediction in mine drainage, groundwater and marine metagenomic sequences downloaded from the NCBI sequence reed archive. By consulting the miRBase repository, 179 miRNAs have been identified as highly probable miRNAs. Our new approach could thus be used for mining metagenomic sequences and finding new and homologous miRNAs.

**Conclusions:**

The paper investigates a computational tool for miRNA prediction in genomic or metagenomic data. It has been applied on three metagenomic samples from different environments (mine drainage, groundwater and marine metagenomic sequences). The prediction results provide a set of extremely potential miRNA hairpins for cloning prediction methods. Among the ensemble prediction obtained results there are pre-miRNA candidates that have been validated using miRbase while they have not been recognized by some of the base classifiers.

## Background

MicroRNAs (miRNAs) are short (∼22 nucleotides), endogenously-initiated non-coding RNAs that control gene expression post transcriptionally, either by the degradation of target mRNAs or by the inhibition of protein translation.

The prediction of miRNA genes is a challenging problem towards the understanding of post transcriptional gene regulation. The two frontier strategies for miRNA prediction are experimental cloning and in silico [1]. However, due to the difficulty of miRNA prediction using experimental techniques, computational approaches have been developed to conquer some of the technical difficulties of the experimental approaches.

The miRNA identification problem is usually defined over pre-miRNAs because their lengths are larger than that of mature miRNAs and, hence, more information can be extracted from their sequences. Moreover, the hairpin stem loop secondary structure of pre-miRNAs is an essential feature used in the computational identification of miRNAs. However, many sequence fragments in a genome have a similar stem-loop hairpin structure, in spite of not being genuine miRNA precursors [2].

Two major computational prediction strategies are considered, either by using homology or by using machine learning methods. Most miRNA prediction methods were developed to find out homologous miRNA in closely related species. These methods use comparative genomics information besides structural features that are extracted from the typical hairpin structures of known pre-miRNAs. ‘Blastn’ adopts the homology principle in miRNA prediction [3].

Comparative genomics is used to filter most of the hairpins that are not conserved in related species. This filtration step makes the method unable to recognize new miRNAs for which there are no known close homologies. Therefore, the attitude turned to focus on machine learning methods to distinguish real pre-miRNAs from other hairpin sequences with similar stem loop features (pseudo-miRNA) [4]. The early machine learning methods used to discriminate real versus pseudo-miRNAs are miRScan [5], miRseeker [6], miRfinder [7] and miRCheck [8].

An amazing extensive wide variety of support vector machine systems have been built, aiming to get better results in predicting miRNAs. The first two of these systems are miR-abela [9] and Triplet-SVM [2].

The miR-able algorithm succeeded to predict between fifty and hundred novel pre-miRNAs [9]. 30% of these have been verified experimentally as real miRNAs.

Triplet-SVM has been prominent due to its simplicity [2]. In this method, a set of features are extracted, and provided to a support vector machine classifier to differentiate between real and pseudo-miRNAs. 90% recognition rate has been achieved.

RNAmicro is a compound prediction method [10]. It first applies a homology strategy to recognize conserved almost-hairpins in a multiple sequence alignment. Then it computes a vector of numerical descriptors from each almost-hairpin that is used by a support vector machine classifier.

Two other systems have been derived from Triplet-SVM approach: MiPred [4], and miREncoding [11]. MiPred annexed two thermodynamical features (Minimum Free Energy MFE, and the P-value), and succeeded in getting better results by using Random Forests instead of SVM. MiREncoding added several new features and tried to enhance the SVM classification performance by using a feature selection algorithm.

Another SVM, miPred [12], improved the accuracy of the previous SVM-classifiers by making extensive use of thermodynamical features. It uses normalized features which are computed on a large number of shuffled versions of a given pre-miRNA. However, this method is not reinforced by biologists due to its lack of biological plausibility. In addition, the normalization process is computationally time consuming.

The microPred [13] is another forceful SVM classifier that obtained more effective results than the previous classifiers due to the use of a negative data set (consisting of ncRNA and pseudo hairpins), new biologically relevant features, feature selection, extensive and systematic training and testing of the classifier system.

Virgo is a viral miRNA precursors prediction method [14]. The method is based on both sequence and structure features that are extracted and fed to an SVM classifier to distinguish pre-miRNA hairpin sequences from pseudo-miRNA hairpin sequences. The method is more efficient than other ab-initio methods for predicting viral and mammalian miRNAs.

EumiR, being an eukaryotic microRNA precursor prediction server, queries multiple sequences to determine if they are true miRNAs or not [15]. EumiR and Virgo share the same prediction principle. Eukaryotic pre-miRNA are used in training EumiR.

YasMiR is, also, an SVM for miRNA identification [16], whose novelty is two-folded: firstly, many of its features incorporate the base-pairing probabilities provided by Mc-Caskill’s algorithm and secondly, its classification performance has been improved by using a similarity (“profile”-based) measure between the training and the testing miRNAs and a set of carefully chosen (“pivot”) RNA sequences.

In the present paper, a computational method is proposed for the identification of miRNA precursors. The method combines the outcomes of four previously developed classification approaches using a neural network, to enable more accurate prediction of miRNAs.

Our method investigates whether a given sequence is a true or pseudo-miRNA using Support vector machines (SVM) and Random Forests (RF), since both of them are optimal binary classifiers.

Our de-novo miRNA prediction method is applied on three metagenomic samples from different environments. The prediction results provide a set of highly probable miRNA hairpins for future laboratory testing. This may lead to the discovery of new miRNA candidates.

Specifically, Triplet-SVM [2], MiPred [4], Virgo [14] and EumiR [15] classifiers are used for miRNAs prediction, and their prediction results are combined using a single hidden layer neural network in the hope of obtaining a more accurate miRNA predictor.

Our ensemble classifier achieved an excellent performance. This encouraged us to rely on it in identifying new miRNA candidates. We identified 106 sequences in the mine drainage metagenome, 55 in the groundwater metagenome and 18 in the marine metagenome as highly probable miRNAs.

This paper is organized as follows. Section ‘Background’ gives an overview of the miRNA prediction techniques. Section ‘Methods’ presents the proposed methodology. Section ‘Results and discussions’ analyses the prediction results. Section ‘Conclusions’ concludes the paper and suggests future work.

## Methods

This section discusses the data sets that are fed into our ensemble classifier. Then, the mechanism of the data preparation is described. Finally, the structure of the adopted approach is explained in details.

### Data sets

Generally, microbiology has concentrated on individual species in pure laboratory. Therefore, the understanding of microbial communities has lagged behind understanding of their individual members. Metagenomics is a new tool to study microbes in the complex communities; where they live and how they interact with their surrounding environments [17,18]. Metagenomics (also known as environmental genomics or community genomics) is the study and analysis of genomes of microbial organisms recovered directly from their natural environments [17,19].

Whole Genome Shotgun sequencing is the procedure of breaking up a target genomic region into many segments, and sequencing them randomly. Through whole-genome shotgun sequencing of collected DNA from environmental patterns, metagenomics has played the role of systematic realization of the nucleotide sequence, followed by analysis of the structure, regulation and function of genes. The primary benefit of metagenomics is that it provides the ability of effectively characterizing the genetic variety existing in samples, without the need for isolation and lab refinement of individual species [18].

In this paper, an ensemble approach is used for miRNA mining in three metagenomic sequences from different environments. These metagenomes (mine drainage, groundwater, and marine metagenomic sequences) have been sequenced in whole-genome shotgun sequencing projects. Details about these projects are available in [20-22].

### Data preparation

Three samples of the considered metagenomes (mine drainage, groundwater, and marine metagenomic sequences): each consisting of twenty contigs from the metagenome; have been randomly selected. As the miRNA prediction problem is usually defined over pre-miRNA and these stem-loop precursors are approximately 60 ∼70 nucleotides [23,24], we developed a Perl script to divide each sample into fragments (70 nucleotides each). Many studies uses the same sample size [10,25]. Each fragment in the sample starts with only one nucleotide shift from the start of the previous fragment to make sure that the miRNA mining covers all possible metagenomic sequences. This yields 97336 sequences for the mine drainage metagenome, 24625 sequences for the ground water metagenome, and 16709 sequences for the marine metagenome. Then, a feature vector; extracted from these fragments; is fed to the ensemble classifier to decide whether it is possibly a miRNA or not.

### The ensemble classifier

The proposed ensemble approach aims to combine the decisions of four miRNA predictors that have been trained on different data and features. The motivation behind the assembling of the classifiers is the better performance and results achieved by consensus predictors and meta-classifiers in bioinformatics analysis that make the implementation of a meta-classifier a good decision for our method. The performance of any classifier is affected by several factors including the size of the training data set, its dimensionality, the number of classes to be differentiated and their mutual separability. Ensemble methods have been devised to reduce over-fitting and improve the performance of individual classifiers by fusing their decisions [26].

Ensemble design is either based on bagging or boosting. In bagging (bootstrap aggregating) ’m’ models are fitted using ’m’ bootstrap samples and combined by averaging or voting. Samples bootstrapping aims at creating diversity in the training data while average/voting aims at improving classification performance. Boosting is implemented either by varying the weights given to the data samples or by forming committees. Boosting is based on the idea that a strong classifier can be constructed from weak classifiers [27]. Our proposed classifier adopts a hybrid scheme. The base classifiers have, originally been trained using different data samples of different dimensionality. Therefore, diversity in the training data is achieved (bagging principle). Also, it belongs to the class of "committees" (boosting principle). Our adopted scheme offers the advantage of being non-linear (because of the sigmoid activation functions in the first layer of the proposed Neural Network).

**Figure 1:**
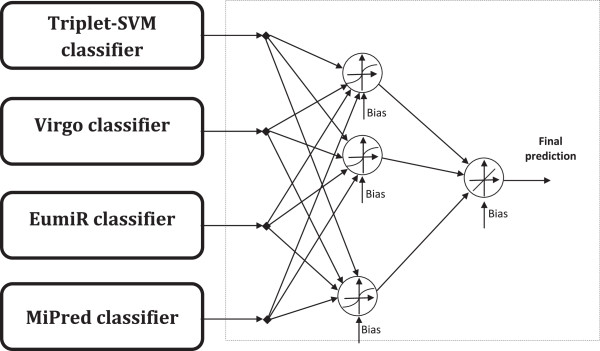
The adopted architecture for miRNA prediction ensemble-based classification approach.

Generally, classifiers differ in the training features, the training data set or the classification method itself. The choice of the four classifiers used in our proposed system is based on these different characteristics. Triplet-SVM, Virgo, EumiR are based on the SVM classification technique while MiPred uses Random Forests classification technique. All of the four classifiers make use of structure- sequence features to categorize true pre-miRNAs and pseudo-miRNAs. However Triplet-SVM and MiPred use the triplet elements definition to represent these features. Moreover, Virgo and EumiR utilize a different definition of these features. In addition to the structure-sequence features, MiPred adds two thermodynamic features. The data used for the training of each of the four classifiers is different. Triplet-SVM and MiPred are based on human pre-miRNAs as positive samples. Virgo relies primarily on viruses pre-miRNAs, and EumiR uses pre-miRNAs from different eukaryotic species. Table 1 summarizes the main characteristics of the classifiers used in the proposed ensemble.In addition to the previously mentioned classifiers, a single hidden layer neural network is used to tune their decisions, as shown in Figure 1. This neural network is trained using supervised learning with the pseudo-inverse technique. The training data set used is different than the ones used in the training of each base classifier. The architecture components are discussed below:

**Table 1:** Main characteristics of the classifiers used in the proposed ensemble

**Classifier**	**Learning algorithm**	**Features**	**Training data**
Triplet-SVM	SVM	A vector of 32 structure-sequence features	Human pre-miRNAs
Mipred	RF	A vector of 32 structure-sequence features, MFE and P-value	Human pre-miRNAs
Virgo	SVM	A vector of 512 structure-sequence features	Viruses pre-miRNAs
EumiR	SVM	A vector of 512 structure-sequence features	Different Eukaryotic pre-miRNA
Ensemble-based	Committee classifier	A vector of 4-dimensions (the outputs from the base classifiers)	Human pre-miRNAs

#### ***Triplet-SVM***

The triplet-SVM classifier has been developed for predicting a query sequence with hairpin structure as a real miRNA precursor or not [2]. Triplet SVM uses a set of features that combines the local contiguous structures with sequence information to characterize the hairpin structure of real versus pseudo-miRNAs.

RNAfold program from the RNA Vienna package has been used to predict the secondary structure of the query sequences [28]. In the predicted secondary structure, Each nucleotide is paired or unpaired, represented by brackets "(" or ")" and dots ".", respectively. The left bracket "(" indicates that the paired nucleotide is near the 5’-end and can be paired with another nucleotide near the 3’-end, which is represented by a right bracket")". The study utilizes "(" for both situations. According to the previous mentioned definition for any 3 adjacent nucleotides, there are 8 possible structure combinations: "(((", "((.", "(..", "(.(", ".((", ".(.", "..(", and"...", which lead to 32 (4∗8) possible structure-sequence combinations, denoted as "U(((", "A((.", etc... This defines the triplet elements. The triplet elements have been used to represent the local structure-sequence features of the hairpin. The occurrence of all triplet elements are counted along a hairpin segment, developing a 32-dimensional vector of features, which is then normalized to be the input vector to the SVM [2].

The SVM classifier is formerly trained depending on the triplet element features of a set of real human pre-miRNAs from the miRNA Registry database [29] as well as a set of pseudo-miRNAs from the NCBI RefSeq database [30]. The training set contains 163 human pre-miRNAs (positive samples) and 168 pseudo-miRNAs (negative samples) randomly chosen.

A 90% accuracy in distinguishing real from pseudo-miRNA hairpins in the human genome and up to 90% precision in identifying pre-miRNAs from other 11 species (including C. briggsae, C. elegans, D. pseudoobscura, D.melanogaster, Oryza sativa, A. thaliana and the Epstein Barr virus) have been achieved.

#### ***MiPred***

MiPred classifier is a Random Forest based method classifier which differentiates real pre-miRNAs from pseudo-miRNAs using hybrid features. The features consist of local structural sequence features of the hairpin with two thermodynamically added features (MFE of the secondary structure that is predicted using the Vienna RNA software package [28] and the P-value that is the fraction of sequences in a set of dinucleotide shuffled sequences having MFE lower than that of the start sequence [31]. P-value is determined using the Monte Carlo randomization test [32]).

MiPred is one of the refinements of Triplet-SVM in which the SVM is replaced by a Random Forests ensemble learning algorithm. The Random Forest prediction model has been trained on the same training data set used by the triplet-SVM-classifier. It achieved nearly 10% greater overall accuracy compared to Triplet-SVM on a new test dataset.

#### ***Virgo***

The Virgo classifier is an efficient prediction classifier that differentiates true pre-miRNAs from pseudo-miRNAs [14]. The classifier has been developed based on sequence structural features. The feature space consists of both sequences and their structural context. A sequence is folded using RNA-fold and the structural context of overlapping triplets is determined. Sequence structure feature space can have 64 possibilities and each nucleotide can have two states, ‘1’ if it is bound and ‘0’ if it is unbound. Thus, such a feature (eg AUG001, AUG010... etc) can have a total of 512 possibilities.

A support vector machine classifier(*SVM*^*light*^)trained on these feature elements is used for efficient distinction between miRNA precursor hairpins and pseudo-miRNA hairpins. The hairpin sequences for the eukaryotes used to train Virgo were derived from miRBase (release 8.0) [33] and the pseudo-miRNA sequences were derived from coding regions of genes with no alternate transcripts. The coding sequences were batch downloaded from Ensembl [34].

Virgo adopts a K-folding like technique for the training phase and selects the classifier that achieves the best specificity. Another advantage of Virgo is using the kernel idea (a Radial Basis function) to find the hyper surface that optimally separates true from pseudo-miRNA hairpins.

Virgo classifier performs better than recently reported methods for machine learning prediction of viral and mammalian pre-miRNAs. The algorithm is fast and efficient and can scale for genome-scale predictions not only on viral genomes, but also on much larger eukaryote genomes.

#### ***EumiR***

EumiR is an eukaryotic microRNA precursor prediction server from IGIB (Institute of Genomics and Integrative Biology), which is able to query multiple sequences to decide whether they are true miRNAs or not. EumiR uses the same principle for prediction of Virgo. RNA-fold is used for predicting the secondary structure of the input sequence. Sequence-structure feature space is determined using the same definition of Virgo.

EumiR utilizes LibSVM package to differentiate pre-miRNA hairpins from pseudo-miRNA hairpins. It is trained using eukaryotic pre-miRNAs from different species as positive samples. EumiR is more efficient in predicting eukaryotic pre-miRNAs, but its efficiency level is not the same for predicting viral microRNAs.

EumiR has better accuracy and sensitivity as compared to mir-abela and BayesmiRNAfind on viral miRNA precursors from miRbase.

EumiR server has more analysis options. It is able to BLAST miRbase, BLAST NCBI, SSEARCH miRbase, predict the secondary structure using RNAfold, predict using BayesmiRNAfind and predict using mir-abela.

#### ***Single hidden layer neural network***

Neural networks are well known for their learning capabilities. Besides, they are model free, i.e., they do not impose any restrictions on the statistical distribution of their input data. The specific Neural-Network-based ensemble works according to the following theorem: A single-hidden layer feed-forward networks with at most N hidden neurons (including biases) can learn N distinct input-output pairs with zero error (It is possible to tolerate a certain amount of error by letting the number of hidden neurons be less than N). This remains true whether the activation function for the hidden neurons is the signum (hardlimit or threshold) or sigmoid (logistic) functions. The activation function of the output neuron(s) is linear. The main advantage of this kind of network is that the hidden layer weights are chosen randomly while the output layer weights can be optimally estimated using the pseudo-inverse solution of an over-determined set of linear equations which is, also, the solution of the least-squares error between the inputs and outputs to/from the neural network. In our proposed ensemble, the inputs (to the ensemble) are the output decisions of four known classifiers; for miRNA prediction; and the output is the corresponding ground truth decision. Therefore, our objective is to learn/calculate the best network weights that map the decisions of the adopted classifiers into a single fused output decision [35,36].

The designed Neural Network has 4 inputs, 3 hidden and 1 output neurons. This network has 19 parameters. 

1. The hidden layer parameters (15 parameters): (*w*_*ik*_) are the weights between the four inputs and the three hidden neurons plus three biases for the hidden neurons.

2. The output layer parameters (4 parameters): (*w*_*kj*_) are the weights between the three hidden neurons and the single output neuron plus the bias of the output neuron.

The inputs to the neural network are the outputs of the classifiers described previously and the teaching output is ‘1’(‘-1’) corresponding to true (false) miRNA. The neural network MATLAB toolbox has been used for modelling and training of the network.Sigmoid activation functions are used for the hidden layer neurons to maintain the non-linearity of the used classifiers. A linear weighted combination of the outputs of the hidden neurons represents the output of the ensemble. This linear combination is produced by using a linear activation function for the output neuron as shown in Figure 1.

The weights of the hidden layer are initialized at random. Since the output neuron is linear, its weight vector can be calculated optimally by solving a set of over-determined linear simultaneous equations using the pseudo-inverse technique. Several random initializations of the weights of the hidden layer have been tried and the best neural network (that achieves the minimum sum of the squares of the errors) has been selected. The optimal parameters of the neural network are shown in Table 2.

**Table 2:** The neural network parameters

**The optimal hidden layer parameters**					
**Neurons**	**Weights**				**Bias**
Neuron1	0.8523	0.7190	0.0156	0.3914	-0.0528
Neuron2	-0.4030	-0.3190	0.7133	0.2558	0.8994
Neuron3	-0.03238	-0.7238	-0.2314	-0.0992	-0.8330
**The optimal output layer parameters**					
**Neurons**		**Weights**			**Bias**
Output		-0.0877	-0.0166	-1.1731	-0.0336

The training dataset consists of 500 known human pre-miRNAs; retrieved from miRBase19 [33]; and 1000 pseudo hairpins; extracted from human RefSeq genes [30].

## Results and discussions

Performance evaluation and prediction results of our proposed ensemble classifier are discussed below.

### Performance evaluation of the ensemble method using the classification statistics

A testing data set consisting of 500 known human pre-miRNAs and 1000 pseudo hairpins - different than those used in training - retrieved from miRBase19 [33] and human RefSeq genes [30]; respectively; has been used for testing the performance of the already trained ensemble classifier.

Four measures are estimated: true positive (TP), true negative (TN), false positive (FP), and false negative (FN). These values are used to assess the performance of the proposed prediction classifier. The considered classification statistics are Accuracy, Sensitivity, Specificity, Precision, F–measure and Negative Predictive value (NPV).

(1) $$\text{Accuracy}=\frac{\text{TP}+\text{TN}}{\text{TP}+\text{TN}+\text{FP}+\text{FN}}*100\%$$

(2) $$\text{Sensitivity}=\frac{\text{TP}}{\text{TP}+\text{FN}}*100\%$$

(3) $$\text{Specificity}=\frac{\text{TN}}{\text{TN}+\text{FP}}*100\%$$

(4) $$\text{Precision}=\frac{\text{TP}}{\text{TP}+\text{FP}}*100\%$$

(5) $$F\;-\text{measure}=2*\frac{\text{Precision}*\text{Sensitivity}}{\text{Precision}+\text{Sensitivity}}*100\%$$

(6) $$\text{NPV}=\frac{\text{TN}}{\text{TN}+\text{FN}}*100\%$$

**Figure 2:**
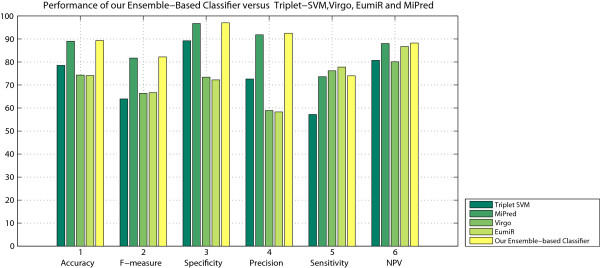
Performance of our Ensemble-based classifier versus Triplet-SVM, MiPred, Virgo and EumiR.

Table 3 compares the performance of our ensemble classifier with the four adopted classifiers. The results confirm that our ensemble classification approach performs well relative to the other adopted approaches.As can be seen from Figure 2, the proposed classifier outperforms the other classifiers for five out of the six adopted performance indices.

**Table 3:** Performance of ensemble-based classifier versus the other adopted classifiers

**Classifier**	**Accuracy**	**F-measure**	**Specificity**	**Precision**	**Sensitivity**	**NPV**
Triplet-SVM	78.5%	63.9%	89.2%	72.6%	57.2%	80.7%
Mipred	89%	81.7%	96.7%	91.8%	73.6%	87.9%
Virgo	74.3%	66.4%	73.4%	58.9%	76.2%	80.1%
EumiR	74.1%	66.7%	72.2%	58.3%	77.8%	86.7%
Ensemble-based Classifier	89.3%	82.2%	97%	92.5%	74%	88.2%

### Performance evaluation of the ensemble method using the receiver operating characteristic

**Figure 3:**
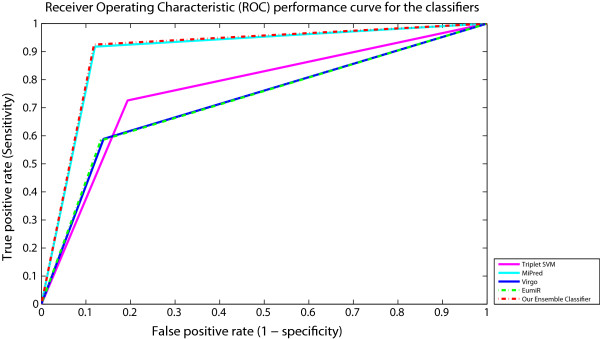
Receiver Operating Characteristic performance curve of the classifiers.

The receiver operating characteristic curve (ROC), is a plot which evaluates the predictive ability of a binary classifier. ROC curves show how the number of correctly classified positive examples varies relative to the number of incorrectly classified negative examples. Figure 3 shows the ROC curve of our Ensemble-based classifier and the four adopted classifiers. Our Ensemble classifier gives a significant performance progress comparing to Triplet-SVM, Virgo and EumiR. Our Ensemble classifier, in general, is consistently better than MiPred classifier. It performs better in the more conservative region of the graph, i.e. it is better at identifying likely positives miRNAs than at identifying likely negatives miRNAs.

The area under the ROC curve (AUC) is a very widely used measure of classifier performance. Table 4 displays the AUC of each adopted classifier versus our ensemble classifier, the 95% confidence interval for sensitivity/ specificity and also the standard error of the AUC (using the method of DeLong et al. for the calculation [37]). The obtained results indicate that our ensemble-based classifier is consistently better.

**Table 4:** ROC- based evaluation metrics of the adopted and designed classifiers

**Classifier**	**AUC**	**95% Confidence interval**	**Standard error**
Triplet-SVM	0.76	0.709 to 0.754	0.0121
Mipred	0.89	0.832 to 0.869	0.0103
Virgo	0.72	0.725 to 0.770	0.0118
EumiR	0.73	0.727 to 0.772	0.0117
Ensemble-based Classifier	0.9	0.836 to 0.872	0.0102

**Figure 4:**
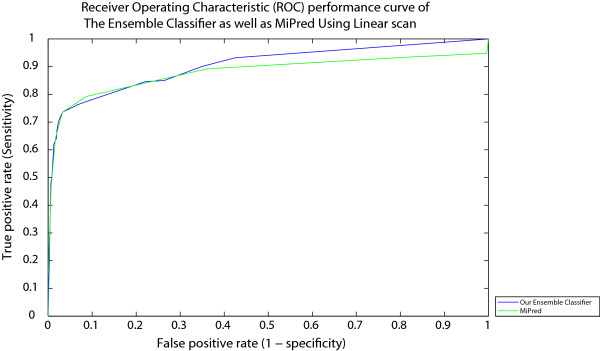
Receiver Operating Characteristic performance curve of Our Ensemble Classifier as well as MiPred using Linear scan.

Figure 4 presents the ROC curves of our ensemble-based as well as MiPred classifiers drawn using Fawcett’s linear scan algorithm for different threshold values [38]. The algorithm generates ROC points given the set of input samples to the classifier as well as the corresponding obtained output scores. For a classifier (like our ensemble); whose output is a score (a numeric value) that represents the degree to which an input sample is a member of a class; each point on the ROC graph can be labelled by the score (threshold) that produces it. Hence, each threshold value (or score value) produces a different point in ROC space. A threshold of + *∞* produces the point (0, 0). As the threshold is reduced, the curve climbs up to the right, ending up at (1, 1) with a zero threshold. Fortunately, we have found that Mipred, also, produces a confidence score for each decision regarding a given input sample.The plot shows that no curve dominates the other over all possible threshold values. Also, The AUC of our ensemble-based classifier is still better than that of “MiPred”. It is important to emphasize, here, on the following idea: the data according to which the ROC curves have been drawn (in Figure 4) belong to the set of data for which “Mipred” has been optimized (of course, this data is different from the data used for training). However, when both classifiers (Mipred as well as the designed ensemble) are exposed to the metagenomic data (which are collected from different environments) one expects that the generalization power of the ensemble should be better (simply because the ensemble consults other classifiers that observe different features of the input samples).

### Prediction results

The proposed classifier has been applied on the metagenomic data sets; described in Section ‘Methods’; and the obtained results are as follows: 106 miRNA candidates have been discovered in the mine drainage metagenome, 55 miRNA candidates have been identified in the ground-water metagenome and 18 miRNA candidates have been predicted in the marine metagenome.

**Figure 5:**
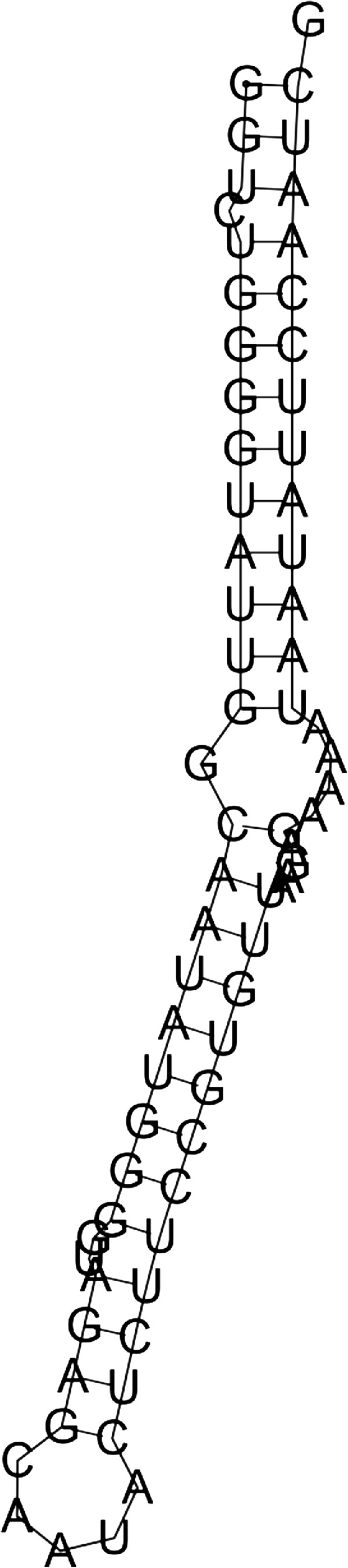
Marine metagenome pre-miRNA candidate.

To validate the predictions, a search for similarity between miRBase miRNA sequences and the predicted sequences has been performed using the BLASTN search algorithm. The miRBase is a database for miRNA sequences, targets, and annotations, that is freely available for online searching at http://microrna.sanger.ac.uk/[39]. The similarity search confirms that our results are valuable. The search demonstrates that there are existing mature miRNAs; that are homologous to the predicted miRNAs sequences in various species. As an example, the secondary structure of the marine metagenome pre-miRNA candidate is shown in Figure 5. This miRNA candidate is similar in homology to gma-miR393f gene that has been experimentally verified as a mature miRNA [40]. (See the predicted sequences in Additional file 1).

Table 5 gives more samples of our prediction results including how many homologous miRNAs are found with the aid of BLASTN in each case. Also, the most trusted homologues which achieve highest similarity scores are listed with their corresponding species.

**Table 5:** Samples of the obtained prediction results

**Sequence**	**Number of**	**Most trusted homologues**	**Species**
	**homologous**		
Marine sequence 1	56	gma-MIR393f and oan-miR-1353	Glycine max and Ornithorhynchus anatinus
Marine sequence 9	28	osa-miR5072 and age- miR-513c-1	Oryza sativa and Ateles geoffroyi
Mine drainage sequence 1	45	ppt- miR1215 and pdi- miR7720	Physcomitrella patens and Brachypodium distachyon
Mine drainage sequence 18	49	pma-miR-138b and osa- miR1851	Petromyzon marinus and Oryza sativa
Mine drainage sequence 29	12	hco- miR-5983 and sme-miR-2167	Haemonchus contortus and Schmidtea mediterranea
Mine drainage sequence 35	29	ppt- miR537d and hma- miR-3005	Physcomitrella patens and Hydra magnipapillata
Mine drainage sequence 41	34	gga- miR-6611 and mtr-miR5037a	Gallus gallus and Medicago truncatula
Mine drainage sequence 53	27	aly- miR3444 and hsa-miR-4440	Arabidopsis lyrata and Homo sapiens
Mine drainage sequence 67	71	lja-miR7526f and cte- miR-96	Lotus japonicus and Capitella teleta
Mine drainage sequence 72	26	cel-miR-90 and dps-miR-2543a-1	Caenorhabditis elegans and Drosophila pseudoobscura
Mine drainage sequence 88	62	hsa- miR-3167 and bdi- miR7711	Homo sapiens and Brachypodium distachyon
Groundwater sequence 1	12	ssc-miR-486-2 and hsa- miR-661	Sus scrofa and Homo sapiens
Groundwater sequence 10	14	csi-miR3950 and cel-miR-87	Citrus sinensis and Caenorhabditis elegans
Groundwater sequence 16	98	mmu-miR-8112 and tgu-miR-2981	Mus musculus and Taeniopygia guttata
Groundwater sequence 23	35	rco-miR156h and hsa-miR-4483	Ricinus communis and Homo sapiens
Groundwater sequence 37	29	osa-miR531 and ggo-miR-760	Oryza sativa and Gorilla gorilla
Groundwater sequence 50	54	hsv1-miR-H17 and mmu-miR-5131	Herpes Simplex Virus 1 and Mus musculus

## Conclusions

A computational tool for miRNA prediction; in genomic or metagenomic data; has been developed. It has been tested on three metagenomic samples from different environments (mine drainage, groundwater and marine metagenomic sequences). The prediction results provide a set of highly probable miRNA hairpins for cloning prediction methods.

The results obtained in this paper are very promising, paving the road for future research in different directions. These directions include miRNA mining in genomic/metagenomic sequences, developing other approaches for ensemble classifiers design, and applying feature selection methods to choose a reduced set of uncorrelated features for miRNA prediction.

## Availability and requirements

The proposed method is a neural network ensemble classifier. The outputs from four known miRNA prediction methods (Triple-SVM, MiPred, Virgo and EumiR); dealing with different miRNA features; are fed into a single hidden layer neural network that is trained to predict the likelihood that an input sample is a miRNA. The source code of each of the considered classifiers is freely accessible [2,4,14,15]. The code for the neural network classifier is available as supplementary file. (See Additional file 1).

The used training and testing data sets consisting of known human pre-miRNAs and pseudo hairpins have been retrieved from miRBase19 [26] and human RefSeq genes [24]; respectively. (See the data sets in Additional file 2).

The approach is applied on metagenomic sequences from different environments (mine drainage, groundwater and marine metagenomic sequences) downloaded from the NCBI sequence reed archive [20-22]. (The Metagenomic Samples are listed in Additional file 3).

106 miRNA candidates have been discovered in the mine drainage metagenome sample, 55 miRNA candidates have been identified in the ground-water metagenome sample and 18 miRNA candidates have been predicted in the marine metagenome sample. (The predicted sequences are listed in Additional file 4).

## Competing interests

We declare that we have no competing interests.

## Authors’ contributions

SHM determined miRNA features, implemented the proposed Ensemble-Based Classification Approach, tested and analyzed the proposed classifier, and wrote the draft manuscript. MH suggested the use of metagenomic sequences, helped in the feature extraction phase and participated in analysing the results; and ASH suggested the proposed ensemble model, guided its development, revised and enhanced the paper presentation. All authors read and approved the final manuscript.

## Supplementary Material

Additional file 1 **Source code.** This file contains the program of the presented classifier. Click here for file

Additional file 2 **Datasets.** This file contains the training and testing datasets. Click here for file

Additional file 3 **Metagenomic samples.** This file contains the metagenomic samples. Click here for file

Additional file 4 **Prediction results.** This file contains all the prediction results for the three metagenomic samples. Click here for file
